# Antibiotic Resistance and Regulation of the Gram-Negative Bacterial Outer Membrane Barrier by Host Innate Immune Molecules

**DOI:** 10.1128/mBio.01541-16

**Published:** 2016-09-27

**Authors:** Samuel I. Miller

**Affiliations:** Departments of Microbiology, Medicine & Genome Sciences, University of Washington, Seattle, Washington, USA

## Abstract

The Gram-negative outer membrane is an important barrier that provides protection against toxic compounds, which include antibiotics and host innate immune molecules such as cationic antimicrobial peptides. Recently, significant research progress has been made in understanding the biogenesis, regulation, and functioning of the outer membrane, including a recent paper from the laboratory of Dr. Brett Finlay at the University of British Columbia (J. van der Heijden et al., mBio 7:e01238-16, 2016, http://dx.doi.org/10.1128/mBio.01541-16). These investigators demonstrate that toxic oxygen radicals, such as those found in host tissues, regulate outer membrane permeability by altering the outer membrane porin protein channels to regulate the influx of oxygen radicals as well as β-lactam antibiotics. This commentary provides context about this interesting paper and discusses the prospects of utilizing increased knowledge of outer membrane biology to develop new antibiotics for antibiotic-resistant Gram-negative bacteria.

## COMMENTARY

The United States Centers for Disease Control and Prevention estimates that each year in the United States, 2 million illnesses resulting in 23,000 deaths are caused by highly antibiotic-resistant bacteria. Many of these infections are caused by Gram-negative bacteria, which have an additional membrane layer termed the “outer membrane.” Gram-negative bacteria cause a wide spectrum of diseases, including urinary tract, bloodstream, airway, venereal, and health care-associated infections. The outer membrane is a largely asymmetric bilayer composed of glycolipid lipopolysaccharides (LPS) and glycerol phospholipids. The outer membrane serves as a barrier for protection against toxic compounds, including antibiotics, whose targets are largely beyond this surface layer (2).

The outer membrane likely evolved, in part, to protect the bacteria against damage from antibiotics produced within multispecies microbial communities, where they function in microbial communication and competition. Therefore, one major function of the outer membrane is to both promote antimicrobial resistance and to interpret bacterial signals from membrane-damaging agents, including antibiotics. In addition to protecting the organism from toxicity, the outer membrane efficiently allows the uptake of soluble nutritional components through specific beta-barrel protein channels, termed “porins,” which are outer membrane protein components with a distinct beta-barrel structure largely only found elsewhere in the outer membrane of eukaryotic mitochondria. The outer membrane barrier can be regulated by environmental signals, including antibiotics, and damage to the outer membrane can be both sensed and repaired. Therefore, the outer membrane can be thought of as an important, regulated organelle protecting the bacteria from toxicity, while allowing the uptake of valuable compounds utilized by the organisms for growth.

Recently Gram-negative bacteria with resistance to commonly used antibiotics, including quinolones, colistins (polymyxins), carbapenems, cephalosporins, and other β-lactam antibiotics, have been isolated from humans with increasing frequency. These bacteria use a wide variety of mechanisms to resist antimicrobial killing, many of which are carried on mobile genetic elements transmitted to other bacteria (2). Since fewer new antibiotics targeting Gram-negative bacteria are in development and organisms that resist all or most clinically useful antimicrobials are already being isolated, drug-resistant infections due to Gram-negative bacteria represent a significant health care concern for the future. Mechanisms of Gram-negative antibiotic resistance include (i) acquisition of enzymes that modify or destroy antibiotics, such as aminoglycoside-modifying enzymes and extended spectrum -lactamases and carbapenemases, (ii) acquisition of enzymes that alter bacterial antibiotic targets, such as lipid A-modifying enzymes conferring resistance to colistins, and (iii) acquisition of mutations in bacterial targets such as topoisomerases, ribosomes, penicillin-binding proteins, and outer membrane porins that alter antibiotic efficacy or uptake (2). Topoisomerases are the target of gyrase inhibitors such as quinolones, ribosomes are the targets of chloramphenicol and streptomycin, and penicillin-binding proteins are important for biosynthesis of peptidoglycan, which makes up the bacterial cell wall and is the target of -lactam antibiotics. Most antimicrobials must traverse the outer membrane barrier to access these targets.

**FIG 1:**
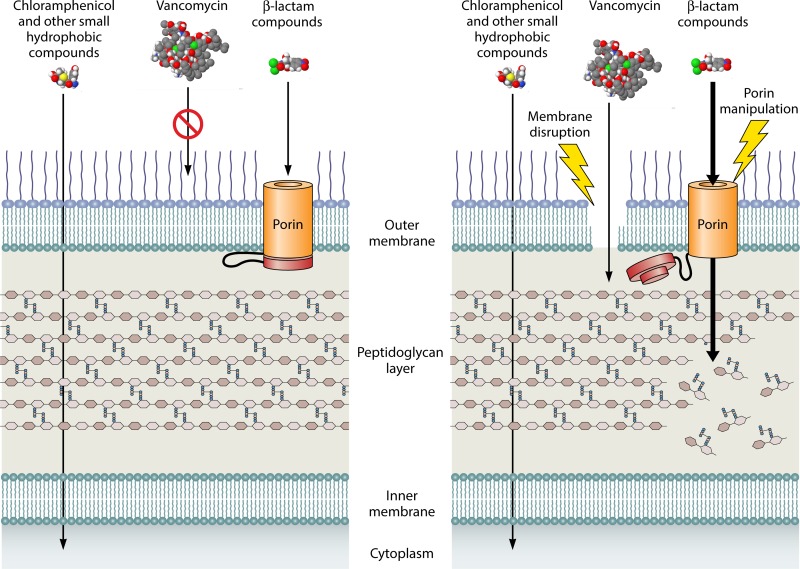
(Left) Diffusion through the outer membrane bilayer allows hydrophobic compounds like chloramphenicol to reach their intracellular targets, but porin activity is required for transport of β-lactam compounds across the outer membrane. (Right) A compound that permeabilizes the outer membrane or dysregulates porin selectivity may allow β-lactam transport and peptidoglycan targeting, broadening the efficacy of our current drug arsenal against Gram-negative bacteria.

Other than polymyxins and other cationic antimicrobial peptides, which disrupt and permeabilize the outer membrane barrier, antibiotics can take one of two pathways to traverse the outer membrane. Many hydrophobic antibiotics, such as chloramphenicol and aminoglycosides, use a diffusion pathway through the lipid components of the outer membrane. In contrast, hydrophilic compounds, such as β-lactam-based antibiotics, move through the outer membrane through porins or selective channels formed by specific beta-barrel proteins, which typically form trimers to create specific hydrophilic pores in the membrane (Fig. 1). Many antibiotics, such as vancomycin, which like β-lactam antibiotics targets the cell wall peptidoglycan, are ineffective against Gram-negative bacteria, simply because they have chemical properties that do not allow them to utilize these pathways to effectively penetrate the outer membrane. Furthermore, resistance mechanisms to specific antibiotics occur when bacteria alter this barrier, either by changing the hydrophobic properties of the membrane or by null mutations in porins, which can create resistance to many β-lactam antibiotics. Loss of porin transport channels has recently resulted in clinically important resistance to imipenem for *Acinetobacter baumanii*, a Gram-negative bacterium that can cause important hospital-acquired infections and for which isolates that are resistant to virtually all clinically useful antibiotics have been recovered. Many uncharacterized *Pseudomonas aeruginosa* isolates from individuals with cystic fibrosis demonstrate significant clinical resistance to tobramycin as a result of loss of outer membrane permeability of the antibiotic. Therefore, the need for new antibiotics for resistant Gram-negative bacteria could be partially obviated by the development of compounds that target the outer membrane barrier and improve the influx of antibiotics, particularly for those antibiotics that are ineffective in penetrating the outer membrane.

The glycolipid components, in particular the lipopolysaccharide (LPS) structure of the outer membrane, can be regulated and are induced in host tissues to promote bacterial virulence (3). This is particularly well studied in *Salmonella* spp. Upon colonization of host tissues, the bacteria are exposed to acidic pH and host antimicrobial peptides, and *Salmonella enterica* serovar Typhimurium modifies all outer membrane components, including protein, LPS, and glycerol phospholipid constituents. In particular the modification of LPS phosphates, which are the target of colistins or polymyxins, leads to resistance to antimicrobial peptides, including polymyxins or colistins, and genes encoding proteins that accomplish one such modification, addition of ethanolamine, have recently been reported as being carried on a mobile plasmid promoting high-level colistin resistance in clinical isolates of *Escherichia coli*. In addition, changes to the lipopolysaccharide and phosphatidylglycerol acylation state can alter membrane hydrophobicity, which alters amphipathic antimicrobial peptide susceptibility and can lead to resistance to hydrophobic antibiotics, such as nalidixic acid.

Now, in a recent article, van der Heijden et al. (5) define another critical *S. Typhimurium* outer membrane permeability alteration that affects microbial virulence characteristics and antibiotic resistance. Their work shows that peroxide treatment, similar to the oxidative stress found in host tissues, alters outer membrane porins and changes sensitivity to reactive oxygen species and uptake of β-lactam antibiotics. They first demonstrate that at least part of the diffusion of peroxide into bacteria depends on porin channels. Resistance to peroxide is important to microbial virulence, because bacteria are exposed to oxidative stress and oxygen radicals produced by phagocytes in host tissues. They then determine that on exposure to hydrogen peroxide, the bacteria open and close specific pores based on alteration in disulfide bond formation within proteins in the periplasm, the space between the outer and inner membranes. They find that oxidation of either the periplasmic domain of an outer membrane porin or another porin-binding periplasmic protein can regulate the porin channel required for uptake of peroxide. Somehow, in a mechanism that remains to be defined, these proteins control the porin channel size of these important components of the permeability barrier, and this control is important for bacterial virulence as a result of resistance to peroxide. The authors then demonstrate that this porin channel change rapidly decreases transport of β-lactam antibiotics. The authors use this information to manipulate the effect on the porins in specific mutants and test them in animal models of infection. They also evaluate the treatment of these infections with cefotaxime, a β-lactam antibiotic transported through the porin pathway. They provide evidence that manipulation of porins is essential for virulence and could increase antibiotic efficacy (Fig. 1), providing a proof of principle that alteration of the outer membrane barrier could provide increased antibiotic efficacy for resistant organisms.

In recent years, there has been considerable progress in the understanding of the biogenesis of outer membrane building block components, including the discovery of pathways for LPS and protein secretion and transport to the outer membrane (1, 4). There has also been considerable progress in understanding the regulation of the outer membrane surface and how diverse bacterial lipids may be regulated within the outer membrane to promote a more robust permeability barrier. Many researchers are optimistic that this information can be utilized to develop new antibiotics that are active on the bacterial surface, since the penetration of antibiotics and the existence of efflux pumps are a significant problem for new antimicrobial development. Therefore, work on the outer membrane, such as that presented in the recently published paper by van der Heijden et al. (5), is essential for the development of antibiotics. It is a reasonable hope that greater knowledge of the Gram-negative bacterial outer membrane can lead to the rational design of effective new antibiotics to treat antibiotic-resistant bacteria.
